# Combined multi-omics approach to identify the key metabolites, key microorganisms and biomarkers correlated with the neutrophil extracellular traps-associated gene TIMP1 in osteoarthritis

**DOI:** 10.3389/fphar.2025.1665228

**Published:** 2025-10-15

**Authors:** Yaoyu Xiang, Jizheng Li, Xidan Hu, Xianguang Yang, Fei Sun, Jing Yang, Weiqing Ge, Tao Zhou, En Song

**Affiliations:** ^1^ Department of Sports Medicine, First Affiliated Hospital of Kunming Medical University, Kunming, Yunnan, China; ^2^ Department of Orthopedics, Yunnan Provincial Hospital of Traditional Chinese Medicine, Kunming, Yunnan, China; ^3^ Clinical Pharmacy Center, First Affiliated Hospital of Kunming Medical University, Kunming, Yunnan, China; ^4^ Department of Orthopedics, Traditional Chinese Medicine Hospital of Luliang County, Qujing, Yunnan, China

**Keywords:** osteoarthritis, neutrophil extracellular traps, TIMP1, animal model, multi-omics

## Abstract

**Background:**

Neutrophil extracellular traps (NETs) contribute significantly to osteoarthritis (OA) pathogenesis; however, the precise molecular interactions remain unclear. This study aimed to identify key NET-associated genes and their correlated metabolites and microbiota in OA through an integrated multi-omics approach.

**Methods:**

Initially, transcriptomic datasets were screened to identify NET-related genes implicated in OA. A rat OA model was established, and the expression of key genes was validated using RT-qPCR, histological analysis, and immunohistochemistry. TIMP1 was selected for further exploration via *in vivo* gene silencing. Subsequently, transcriptomics, metabolomics, and 16S rRNA sequencing were performed on serum, cartilage, and fecal samples from experimental animals. Differentially expressed genes (DEGs), microbiota, and metabolites associated with TIMP1 were identified through integrated bioinformatics analyses. Correlation analyses across omics data layers were conducted to pinpoint biomarkers, key metabolites, and microbial taxa.

**Results:**

ITGB1, ITGB2, MMP9, and TIMP1 emerged as key NET-associated genes, with TIMP1 being selected as the primary target. TIMP1 silencing significantly alleviated inflammatory responses and cartilage degradation in OA rats. Multi-omics analyses identified 6 biomarkers, 9 key metabolites (e.g., FAHFAs, 12-HETE, MTA, xanthosine), and 1 key microbial genus (Muribaculaceae) strongly correlated with TIMP1 expression. These molecular entities were enriched in pathways related to lipid metabolism, nucleotide turnover, immune regulation, and gut-joint crosstalk.

**Conclusion:**

TIMP1 acts as a pivotal regulator in OA, influencing inflammation, cartilage remodeling, metabolic pathways, and gut microbiota composition. This study provides novel mechanistic insights and potential therapeutic targets for OA.

## 1 Introduction

Osteoarthritis (OA) is the most prevalent chronic joint disease, characterized by progressive degeneration of articular cartilage, subchondral bone remodeling, and synovial inflammation, which together lead to joint pain, stiffness, and functional limitation (Tang et al., 2025). Affecting over 595 million individuals globally, osteoarthritis presents a growing public health challenge, particularly in underserved populations and joints, with disparities in prevalence, disability, and treatment outcomes continuing to emerge (Minnig et al., 2024). OA imposes a significant personal and societal burden, yet effective disease-modifying therapies remain unavailable. There is an urgent need to better understand its pathogenesis to identify actionable therapeutic targets. Accumulating evidence now supports OA as a multifactorial disease driven by genetic, mechanical, metabolic, and inflammatory factors (Wood et al., 2023; Binvignat et al., 2024).

In recent years, neutrophil extracellular traps (NETs) have attracted growing attention for their role in chronic inflammation and autoimmune diseases. NETs are web-like structures composed of decondensed chromatin and antimicrobial proteins—such as neutrophil elastase and citrullinated proteins—released by activated neutrophils. Under physiological conditions, they function in innate immune defense by capturing and killing pathogens (H. Wang et al., 2024). However, under pathological circumstances, NETs can contribute to tissue injury and inflammation through multiple mechanisms. Specifically, NET-derived components can directly degrade cartilage matrix and activate synovial fibroblasts and adaptive immune responses, thereby amplifying joint inflammation (Carmona-Rivera et al., 2020). Although NETs have been extensively studied in autoimmune conditions like rheumatoid arthritis, emerging evidence suggests their involvement in osteoarthritis (OA) as well. For instance, increased neutrophil infiltration and elevated levels of anti-HSP60 autoantibodies have been observed in OA synovial fluid, indicating local immune activation possibly driven by NETs (Corsiero et al., 2024). These findings highlight the clinical urgency to better understand NETs in OA, as they may contribute to inflammation, cartilage degradation, and symptom progression in affected patients. Therefore, elucidating the molecular mechanisms linking NETs and OA, and identifying reliable biomarkers, are crucial for developing precision therapies and improving patient outcomes (Courties et al., 2024).

Multi-omics integration—incorporating microbiome, metabolomics, and transcriptomic data—has become an essential strategy for elucidating the complex mechanisms underlying multifactorial diseases such as OA. Unlike single-omics approaches, which often yield fragmented molecular insights, multi-omics allows for concurrent investigation of cross-system interactions, offering a more comprehensive understanding of disease biology (Chen et al., 2023). By integrating host gene expression, metabolic profiles, and microbial composition, researchers can pinpoint specific microbial taxa, metabolic pathways, and gene regulatory networks implicated in joint inflammation, cartilage degradation, and immune dysregulation (Deng et al., 2025; Wang Y. et al., 2024). For instance, short-chain fatty acids derived from gut microbes can modulate host immune signaling and reshape the joint microenvironment. Transcriptomic analyses further identify key regulatory genes and pathways activated in response to microbial and metabolic cues.

This cross-validation across omics layers enhances biomarker discovery and increases analytical robustness.

Moreover, multi-omics approaches can reveal novel molecular signatures not observable in isolated datasets but uncovered through synergistic data layering, offering unique insights into pathophysiological mechanisms and therapeutic targets (Y. Wang et al., 2024). Such integrative strategies hold promise for developing personalized, mechanism-based interventions for complex diseases like OA.

In this study, we systematically integrated transcriptomic, metabolomic, and microbiome data to investigate the role of NET-associated genes in OA. After identifying candidate genes through multi-step bioinformatics screening, we established a rat OA model and performed gene silencing experiments *in vivo*. Multi-omics analyses of serum, cartilage, and fecal samples were then conducted to explore molecular changes associated with gene modulation. This integrative approach provides novel insights into OA pathogenesis and reveals potential biomarkers and therapeutic targets.

## 2 Methods

### 2.1 Data collection

The mRNA data in GSE114007 (GPL11154 and GPL18573) and GSE57218 (GPL6947) datasets associated with osteoarthritis (OA) were acquired from the GEO database (https://www.ncbi.nlm.nih.gov/geo/). The GSE114007 dataset contained 20 articular cartilage samples of OA and 18 normal articular cartilage samples. The GSE57218 dataset contained 33 articular cartilage samples of OA and 7 normal articular cartilage samples. Additionally, 137 neutrophil extracellular traps-related genes (NRGs) were extracted from the literature for further analysis.

### 2.2 Differential expression analysis

The ComBat algorithm in sva package was utilized to remove batch effect within the GSE114007 dataset. Thereafter, differentially expressed genes (DEGs) within OA and normal samples in the GSE114007 dataset were identified by the limma package (v 3.48.3), with adjusted *P*-value (adj.*P*) <0.05 and |log_2_ Fold Change (FC)| >1. This threshold is conventionally used to reduce batch-related noise, high-depth RNA-seq data provide greater dynamic range and thus allow reliable detection of moderate (≥1.4-fold) expression changes (Newton et al., 2025). Volcano map and heat map of differentially expressed genes (DEGs) were plotted via ggplot2 package (v 1.0.12) and pheatmap (v 1.0.12), respectively.

### 2.3 Recognition and analysis of candidate genes

To identify NRGs that played an important role in OA, the intersection of NRGs and DEGs was taken as candidate genes by the ggvenn package (v 1.0.12). Functional annotation of candidate genes was investigated utilizing clusterProfiler package (v 4.0.2) to explore Gene Ontology (GO) functions and Kyoto Encyclopedia of Genes and Genomes (KEGG) pathways (*P* < 0.05).

### 2.4 Determination of key genes and gene set enrichment analysis (GSEA)

Next, candidate genes were entered into STRING database (http://string-db.org), and a confidence score of >0.7 was set. For further selection of candidate genes, results with removing isolated genes were then imported into Cytoscape (v 3.7.1), and the degree algorithm in the cytohubba plugin was applied to calculate gene degree score (degree score >2). Subsequently, the SVM-REF algorithm was carried out by the e1071 package (v1.7-14) to identify SVM-REF feature genes with the highest model accuracy. Concurrently, Boruta algorithm was carried out by Boruta package (v 8.0.0) to identify Boruta feature genes. The intersection genes obtained from two machine learning algorithms were taken as signature genes. Wilcoxon test was applied to test the difference in expression of signature genes in OA samples and control samples in GSE114007 and GSE57218 datasets, respectively (*P* < 0.05). At the same time, ROC analysis was executed relying on signature genes in GSE114007 and GSE57218 datasets, and area under the curve (AUC) values were computed (AUC >0.7). Genes that had consistent expression trends and marked disparities with AUC value surpassed 0.7 in two datasets were defined as key genes.

To explore biological pathways took part in key genes w ere analyzed, and GSEA was executed. In GSE114007 dataset, Spearman correlation analysis was performed within each key gene and all genes. Genes were then ranked from the largest to the smallest by the correlation coefficient. Subsequently, the clusterProfiler package (v 4.0.2) was employed for GSEA (|normalized enrichment score (NES)| >1, *P* < 0.05). The KEGG gene set within the msigdbr package was applied as a background gene set.

### 2.5 Construction of rat model and sample collection

In total, 24 male Sprague-Dawley (SD) rats, aged 6–8 months, were derived from Beijing Sibeifu Bio-Technology Co., Ltd. (Production License No.: SCXK (Beijing) 2019-0010; Use License No.: SYXK (Dian) K2020-0006). The initial body weights of the animals ranged from 246.7 g to 288.9 g, with a mean of 262.5 ± 8.6 g prior to group allocation. Rats were randomly divided into 4 groups: control group with sham surgery (6 rats) (group A), OA group (6 rats) (group B), TIMP1 silencing group (6 rats) (group C, OA + recombinant adeno-associated viral (rAAV)-sh-TIMP1 group), and mock group (6 rats) (group D, OA + rAAV-sh-NC group). This sample size was selected based on prior OA animal model studies using similar omics and histological outcome measures. Although formal power analysis was not conducted due to the exploratory nature of multi-omics investigations and the lack of effect size data, a sample size of six rats per group has been widely adopted and shown to be sufficient to detect biological differences while balancing ethical considerations and resource constraints (Chen et al., 2025). All animals were housed in a specific pathogen-free (SPF) animal facility under standardized environmental conditions: controlled temperature (24 °C ± 2 °C), relative humidity (50%–60%), and a 12-h light/dark cycle. Rats were given free access to water and standard maintenance chow (commercial diet from Beijing KeAo XieLi Feed Co., Ltd.; product code: SPF-grade maintenance feed for laboratory rats, GB14924.3-2010) (Beijing, China). All animals were acclimatized for 12 days prior to the start of the experiment. Bedding was changed regularly, and animal health was monitored daily in compliance with institutional and national animal welfare guidelines. All rats were housed under controlled temperature conditions with *ad libitum* access to food and water. After 12-day adaptation period, surgical induction of OA was performed by medial meniscus resection in the right knee, while the control group underwent sham surgery with joint exposure only. As for the OA group, OA + rAAV-sh-TIMP1 group, and OA + rAAV-sh-NC group, the meniscus of the knee joints of the rats was resected after anesthesia. In contrast, in the control group, only the knee joints of rats were exposed and then sutured. On the 7th day after surgery, rats in OA + rAAV-sh-TIMP1 group and OA + rAAV-sh-NC group were respectively injected with 20 μL rAAV vectors once a week for 6 consecutive weeks. The body weights of rats were recorded every week after surgery.

After rats in each group were anesthetized, blood samples, articular cartilage samples, and faecal samples from all rats were collected for subsequent analysis. Blood samples were allowed to stand at 4 °C overnight to acquire serum.

### 2.6 Enzyme-linked immunosorbent assay (ELISA)

To assess inflammatory response in animal models, the content of IL-1β (Meimian: MM-0047R2, Beijing, China) and TNF-α (Meimian: MM-0180R2, Beijing, China) in the serum of rats was detected employing an ELISA kit. Instructions were followed to operate. Optical density (OD) values were measured at 450 nm by a microplate reader (BioTek: EIx800, Vermont,United States of America) within 15 min. Results from ELISA were exported to Excel, and then imported into Graphpad Prism 5 for statistical analysis and presentation (t-test, *P* < 0.05).

### 2.7 Toluidine blue staining, hematoxylin-eosin (H&E) staining, and immunohistochemical (IHC) analysis

To evaluate whether the animal model had been successfully constructed, a series of experiments were executed on articular cartilage tissue. Specifically, tissues were fixed in 4% paraformaldehyde. After that, they were infiltrated with paraffin and embedded. Subsequently, tissue sections were subjected to toluidine blue staining, H&E staining, and IHC analysis (Feng et al., 2025). For immunohistochemical analysis, 3 μm paraffin sections were incubated with primary antibodies against Collagen II (Huabio, ER1906-48, Hangzhou, China; 1:100), MMP3 (Affinity, AF0217, Liyang, China; 1:50), MMP13 (Bioss, bs-10581R, Beijing, China; 1:100), and TIMP-1 (Bioss, bs-4600R, Beijing, China; 1:100), all diluted in 2% BSA. Antigen retrieval was performed using citrate buffer (pH 6.0), followed by blocking with 5% normal serum. Sections were incubated with the primary antibodies overnight at 4 °C, followed by incubation with horseradish peroxidase (HRP)-conjugated secondary antibodies and visualization using DAB. Counterstaining was carried out with hematoxylin. Images were captured using an inverted microscope (Nikon, TS2), and quantitative analysis of immunopositive staining was performed using Image-Pro Plus software. All staining procedures were conducted under uniform conditions. Quantitative results were statistically analyzed using GraphPad Prism 5.0.

### 2.8 Reverse transcription quantitative polymerase chain reaction (RT-qPCR) analysis

A total of 6 normal tissue samples, 6 OA tissue samples, 6 OA + sh-NC tissue samples, and 6 OA + sh-TIMP-1 tissue samples were taken from rats. In addition, total RNA was extracted from 5 pairs of tissue samples by TRIzol reagent (Vazyme, Beijing, China). Subsequently, RNA concentrations were measured by NanoPhotometer N50. Secondly, mRNA was transcribed to synthesize cDNA employing Hifair^®^ Ⅲ 1st Strand cDNA Synthesis SuperMix for qPCR kit (Yishen, Shanghai, China). Next, above reverse transcription product, cDNA, was diluted with RNase/DNase-free reagents. Finally, RT - qPCR analysis was carried out on a CFX96 real-time PCR detection system (BIO-RAD, Hercules, CA, United States of America). Collected data underwent analysis utilizing the well-established 2^−ΔΔCt^ method, employing GAPDH as a reference gene for normalization. Finally, Graphpad Prism 10 was employed to plot and calculate the *P*-value.

### 2.9 Metabolomics sequencing and data preprocessing

Metabolomic sequencing was conducted on serum samples of rats in groups A, C, and D. Collected samples were extracted with 50% methanol buffer. Briefly, 50% methanol was added to serum, vortexed for 1 min, and then incubated at room temperature for 10 min. Then, the extraction mixture was precipitated overnight at −20 °C. After that, after centrifuging at 4,000 g for 20 min, supernatant was transferred to a new 96-well plate. Before LC-MS analysis, samples were stored at −80 °C. In addition, 10 μL of each sample was taken out to prepare a mixed QC sample. All samples were acquired by an LC-MS system following machine orders.

Chromatographic separations were executed employing an ultra-performance liquid chromatography (UPLC) system (SCIEX, Framingham, MA, United States). An ACQUITY UPLC T3 column (100 mm * 2.1 mm, 1.8 µm, Waters, Milford, MA, United States) was applied for reversed-phase separation. A high-resolution tandem mass spectrometer, TripleTOF5600plus (SCIEX, Framingham, MA, United States) was applied to detect metabolites eluted form the column, and the mode of data acquisition was Information Dependent Acquisition (IDA) mode. Pearson correlation analysis was conducted on abundance values of each QC sample to evaluate repeatability of metabolite detection. Next, raw files were imported into XCMS software for peak extraction and peak alignment to attain original abundance information of each metabolic ion in the samples. Then, metabolites were annotated through HMDB database, the KEGG database and an in-house metabolite secondary spectral library. Subsequently, QC was carried out on metabolites by metaX software. Samples with 80% missing data or QC samples with 50% missing data were excluded. Then, median normalization was employed for data normalization, a minimum imputation method was employed to fill in missing data values. Finally, metabolite data were quantified. Partial Least Squares Discriminant Analysis (PLS-DA) and permutation tests were adopted to evaluate inter-group differences among the three groups, as well as within groups D and A, and within groups D and C. A clustering heatmap was drawn to display the abundances of metabolites.

### 2.10 Confirmation and enrichment analysis of differential metabolites

PLS-DA and Student’s t-test (with metabolite intensities log2-transformed prior to testing) (thresholds: VIP >1, *P* < 0.05 and |log2FC| ≥0.2630344) were conducted on groups D and A to ascertain differentially expressed metabolites (DEMs), which were named as DEMs1. Volcano plot of DEMs1 was generated, and an expression heatmap of the top 30 DEMs1 was drawn. Subsequently, enrichment analysis on DEMs was carried out. Results were displayed by bar chart. The same analysis was conducted within groups D and C to gain DEMs2. The log2 transformation stabilized variance and improved the approximation to normality. The threshold of |log2FC| ≥0.263 (1.2-fold change) combined with VIP >1 is commonly adopted to account for the inherently lower dynamic range of metabolite quantification (Meng et al., 2024; Zhao et al., 2025).

### 2.11 16S rRNA gene sequencing and data preprocessing

16S rRNA gene sequencing was undertaken on fecal samples of rats in groups A, C and D. Firstly, DNA of fecal samples was extracted depending on kit (OMEGA Stool DNA Kit, Omega Bio-Tek, Norcross, GA, United States). Subsequently, PCR amplification (with 35 cycles) was carried out based on total DNA. PCR products were identified by 1.5% agarose gel electrophoresis. Then, target fragments were recovered through kit (AxyPrep PCR Cleanup Kit, Axygen Biosciences, Union City, CA, United States). The PCR product underwent further purification with the help of Quant-iT PicoGreen dsDNA Assay Kit (Thermo Fisher Scientific, Waltham, MA, United States). The library was quantified on Promega QuantiFluor fluorescence quantification system. Finally, sequencing was carried out on an Illumina sequencing platform 250 PE, depending on standard operation procedures. To normalize original data, splicing, quality control, and chimera filtering were carried out employing overlap. The DADA2 was invoked to perform deduplication and denoising, splicing, and chimera removal. Amplicon Sequence Variants (ASVs) were employed to construct an Operational Taxonomic Units (OTU)-like table. QIIME2 was employed to remove chimeras and generate a feature table. Finally, the ggvenn package was employed to create a Venn diagram to visualize the number of ASVs in each group.

### 2.12 Diversity analysis and abundance analysis of gut microbiota

In order to investigate microbiota richness and diversity among the three groups, alpha diversity of microorganisms was computed. Alpha diversity indices included Chao 1, Observed species, Goods_coverage, Pielou-e, Shannon, and Simpson indices. Sequencing depth in the overall level and group level of microorganisms was evaluated through rarefaction curves based on these 6 indices. Next, to explore species differences among groups, Beta diversity was investigated among groups A, C, and D through Principal coordinates analysis (PCoA), Nonmetric Multidimensional Scaling (NMDS), Analysis of similarities (ANOSIM), and Analysis of Variance applying Distance Matrices (Adonis). Both ANOSIM and Adonis (PERMANOVA) are permutation-based, distribution-free methods that do not rely on normality or homogeneity of variances. In light of the ASV abundance table, to assess differences in species composition structure and species diversity among samples, PCoA was carried out in light of four metrics: unweighted unifrac, weighted unifrac, jaccard, and Bray-Curtis. NMDS was utilized to evaluate species composition of samples based on the ordination sequence of the distance matrix (stress <0.2). Anosim analysis was on the basis of four metrics of unweighted unifrac, weighted unifrac, jaccard, and Bray-Curtis to test whether within-group differences markedly exceeded within-group differences, thus judging whether grouping was meaningful (R > 0, *P* < 0.05). Adonis examined the degree of explanation of sample differences by different grouping factors through the distance matrix and utilized permutation tests to assess its significance (*P* < 0.05).

### 2.13 Determination and function prediction of differential microorganisms

In addition, annotations were made by SILVA (Release 138, https://www.arb-silva.de/documentation/release-138/) and NT-16S (https://www.ncbi.nlm.nih.gov/) databases, and the species with the top 30 relative abundances at the genus level were presented through stacked bar charts, heatmaps, and clustering diagrams. Subsequently, to further identify differential microorganisms in A group and D group, differential microorganisms 1 were selected by linear discriminant analysis (LDA) scores and linear discriminant analysis effect size (LEfSe) analyses at the family, genus, and species level (*P* < 0.05, LDA scores >3), a commonly used threshold in microbiome studies to ensure both statistical and biological relevance (Segata et al., 2011). KEGG pathways of differential microorganisms 1 were predicted by PICRUSt2 (*P* < 0.05). The abundance of differential microorganisms 1 at the genus level within groups (D vs. A) was later demonstrated by heatmaps. The above analysis was also conducted within groups D and C, and differential microorganisms 2 were ascertained.

### 2.14 Transcriptome sequencing and data preprocessing

Total RNA of cartilage tissues (groups A, C, and D) was extracted using TRIzol (Invitrogen, CA, United States) and quantified for number and purity by NanoDrop ND-1000 (NanoDrop, Wilmington, DE, United States). Then, the integrity of RNA was detected by Bioanalyzer 2100 (Agilent, CA, United States) and verified by agarose gel electrophoresis. Assay criteria were concentration >50 ng/μL, RNA integrity index (RIN) >7.0, OD260/280 >1.8, and total RNA >1 μg. The mRNA containing polyadenylic acid (PolyA) was specifically captured by Dynabeads Oligo (dT) (Thermo Fisher, Cat. 25-61005, CA, United States). Captured mRNA was fragmented at 94 °C (5–7 min), applying the magnesium ion fragmentation kit (NEBNext^®^ Magnesium RNA Fragmentation Module, Cat. E6150S, United States). Then, cDNA was synthesized by reverse transcription with reverse transcriptase (Invitrogen SuperScript™ II Reverse Transcriptase, Cat. 1896649, CA, United States). Double-stranded DNA was synthesized applying *E. coli* DNA polymerase I (NEB, Cat. m0209, United States), RNase H (NEB, Cat. m0297, United States) and dUTP solution (Thermo Fisher, Cat. R0133, CA, United States). Subsequently, an A base was added to both ends of it, and its fragment size was screened and purified applying magnetic beads. Double-stranded DNA was digested with UDG enzyme (NEB, Cat. m0280, MA, US), and then PCR was carried out (denaturation at 95 °C for 3 min, 8 cycles of denaturation at 98 °C for 15 s, annealing at 60 °C for 15 s, extension at 72 °C for 30 s, and finally extension at 72 °C for 5 min) to attain a library with a fragment size of 300 bp, with a tolerance of ±50 bp. Finally, sequencing was undertaken depending on standard operation applying illumina Novaseq™ 6000. 2 × 150 bp paired-end sequencing (PE150) was chosen as sequencing mode. Raw data were filtered by Trimmomatic software (v 0.39). Subsequently, filtered data were aligned with reference genome (*Rattus norvegicus*.107) through Hisat2 software (v 2.2.1). Gene expression data was extracted by FeatureCount software (v2.0.3) (parameters: -t exon -g gene_name) and then data was converted into Fpkms format. Expression level of each sample was presented by box plots. Finally, PCA was employed to cluster three groups of samples and remove outliers.

### 2.15 Confirmation and enrichment analysis of differentially expressed genes

Ground on transcriptome sequencing data, differentially expressed genes (DEGs) in groups D and A were identified by DESeq2 package (v 1.34.0) and labeled as DEGs1 (adj.*P* < 0.05 and |log_2_FC| >0.5). Volcanomap of DEGs1 was generated by ggplot2 packages (v 3.5.1) and top 10 up and downregulated genes (depending on log_2_FC) were labeled. Heatmap was drawn by ComplexHeatmap packages (v 2.12.1) to show expression patterns of these 20 genes. To explore biological functions and signal pathways that DEGs1 were participated in, clusterProfiler software package (v 4.0.2) was utilized to conduct GO function and KEGG pathway enrichment analyses (P < 0.05). Same analysis was undertaken within groups D and C and acquired DEGs2. The threshold of |log2FC| >0.5 was chosen as a balance between biological sensitivity and statistical robustness in RNA-seq data.

### 2.16 Gene, microorganisms and metabolite correlation analysis

Intersection of DEMs1 and DEMs2, differential microbial genus 1 and differential microbial genus 2, as well as DEGs1 and DEGs2, was taken to acquire candidate metabolites, candidate microbial genera and candidate biomarkers. Subsequently, the mixOmics package (v 6.22.0) was applied to conduct correlation analysis on candidate metabolites, candidate microbial genera, and candidate biomarkers in overall, and then key metabolites, key microbiota, and biomarkers were ascertained (|cor| >0.8). After that, the expression patterns of the three were displayed through a heatmap. In light of key metabolites, key microbiota, and biomarkers, the factoextra package (v 1.0.7) was employed to perform PCA on three groups (A, C, and D) to analyze the expression specificity of key metabolites, key microbiota, and biomarkers. Finally, Spearman analysis was carried out to analyze correlations among key metabolites, key microbiota and biomarkers, as well as correlations within TIMP1 and key metabolites, key microbiota and biomarkers (|cor| >0.3, *P* < 0.05), and FDR correction was applied using the Benjamini–Hochberg method to control for multiple comparisons (Benjamini and Yosef, 1995).

### 2.17 Statistical analysis

All analyses were performed in R version 4.2. Each rat was treated as an independent experimental unit, with random group allocation and separate sampling to ensure independence of observations. Normality was examined with the Shapiro–Wilk test and homogeneity of variances with the Levene or Brown–Forsythe tests. When assumptions were not met, data were log-transformed and re-evaluated; metabolite intensities had already been log2-transformed to stabilize variance. If assumptions were satisfied, one-way ANOVA followed by Tukey’s test was applied. Welch’s ANOVA with Games–Howell was used for unequal variances, and the Kruskal–Wallis test with Dunn’s test was used when normality was violated. Two-group comparisons used Student’s t-test, Welch’s t-test, or the Mann–Whitney U test, as appropriate. The Benjamini–Hochberg method was applied to control the false discovery rate for multiple testing. Differential metabolites were defined by variable importance in projection values greater than 1, P values less than 0.05, and an absolute log2 fold change of at least 0.263. Microbiome β-diversity was assessed with ANOSIM and PERMANOVA, which are permutation-based and distribution-free methods not reliant on normality or homoscedasticity. The specific statistical test used is indicated in each figure legend.

## 3 Results

### 3.1 There were 29 candidate genes ascertained

**FIGURE 1:**
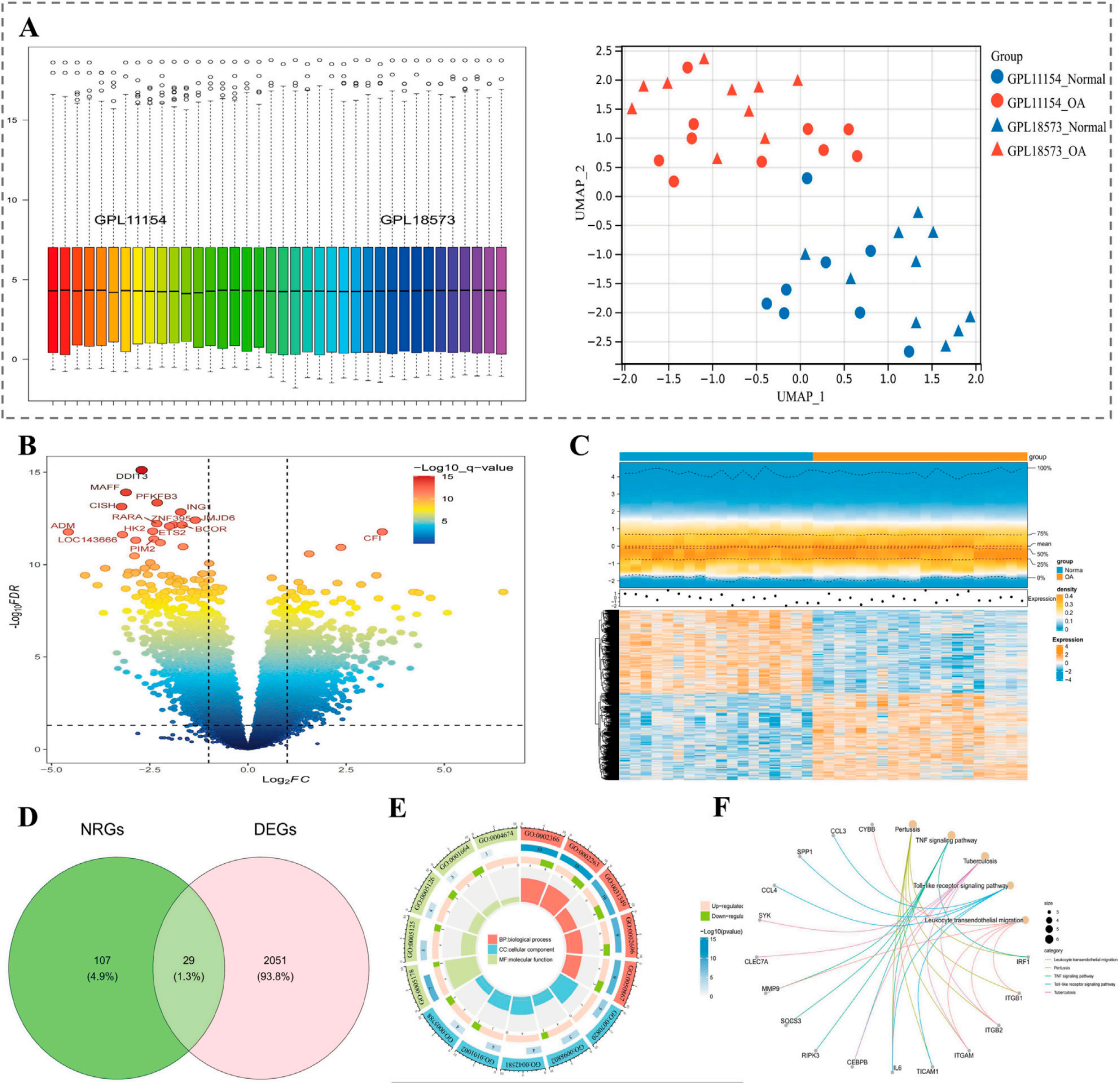
Identification of candidate NET-related genes in OA. **(A)** Boxplot of sample distributions after batch effect removal and UMAP plot showing group separation. **(B)** Volcano plot of DEGs in OA samples; top 15 genes labeled. **(C)** Heatmap of the top 10 up- and downregulated genes. **(D)** Venn diagram showing the intersection of DEGs and NRGs (29 genes identified). **(E)** GO enrichment analysis of candidate genes, including BP, CC, and MF categories. **(F)** KEGG enrichment of the top 5 signaling pathways.

In GSE114007 datasets, after removing batch effect, overall data became relatively stable, and data of the OA group and control group were distinguishable (Figure 1A). There were 2080 DEGs ascertained, which included 1064 up- and 1016 downregulated genes (adj.P < 0.05 and |log_2_FC| >0.5). The top 15 DEGs (P value) were labeled in volcano plot (Figure 1B). Heatmap presented expression patterns of top 10 up/downregulated genes in samples (Figure 1C). Finally, 29 candidate genes were gained by taking the intersection of 2080 DEGs and 136 NRGs (Figure 1D). GO functional annotation of candidate genes revealed enrichment to 798 biological processes (BPs, e.g., macrophage activation), 44 cellular components (CCs, e.g., tertiary granule), and 46 molecular functions (MFs, e.g., integrin binding), with graphs illustrating the top 5 functions (by number of enriched genes) for each part (Figure 1E). Also, 45 KEGG signalling pathways were enriched (e.g., TNF signaling pathway). The graph shows the top 5 pathways of results (ground on *P* value) (Figure 1F).

### 3.2 ITGB1, ITGB2, MMP9, and TIMP1 were considered key genes

**FIGURE 2:**
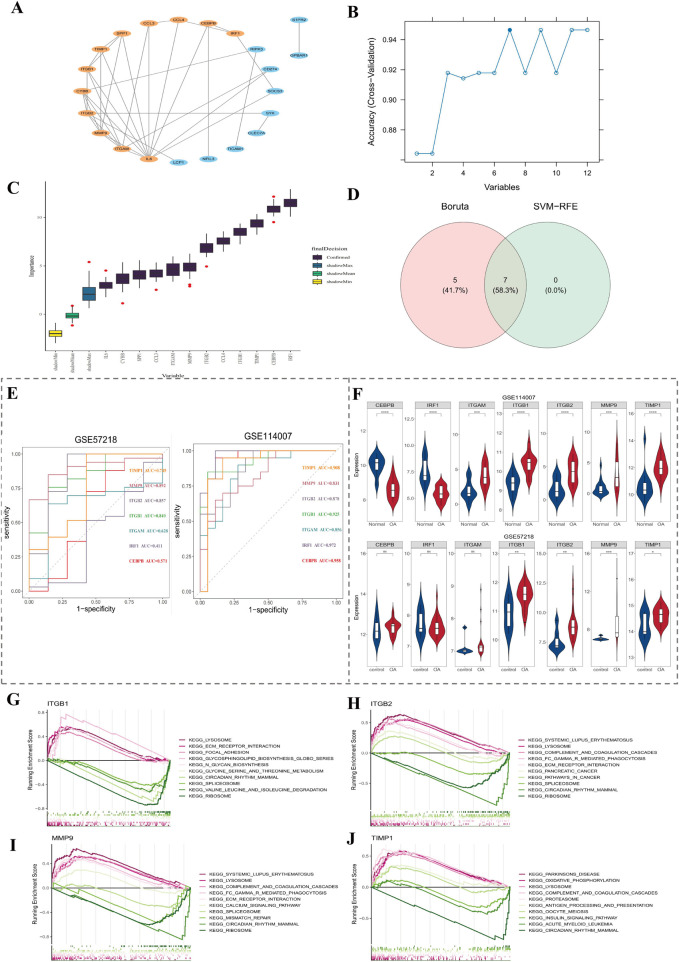
Screening and validation of key genes. **(A)** PPI network analysis identified 12 genes based on degree score. **(B,C)** Seven signature genes were identified using SVM-RFE and Boruta algorithms. **(D)** Venn diagram shows overlapping genes between two machine learning methods. **(E)** ROC curve analysis of 7 candidate genes in GSE114007 and GSE57218 datasets. **(F)** Expression levels of candidate genes across the OA and control groups. **(G–J)** GSEA enrichment of ITGB1, ITGB2, MMP9, and TIMP1; the top 5 up- and downregulated pathways based on NES value are shown.

After removing 7 isolated genes in PPI network, 12 genes were screened out depending on degree score (Figure 2A). Subsequently, 7 SVM-RFE feature genes were ascertained from 12 genes by the SVM-RFE algorithm (Figure 2B). Meanwhile, 12 Boruta feature genes were output in Boruta (Figure 2C). After taking intersection of two machine learning algorithms, 7 signature genes were derived (Figure 2D). Finally, 4 key genes (ITGB1, ITGB2, MMP9, and TIMP1) were identified through ROC curve (Figure 2E) and expression level verification (Figure 2F).

Afterwards, in order to explore signal pathways linked to key genes, GSEA enrichment analysis was carried out. Among them, ITGB1 was enriched in 26 pathways, such as focal adhesion (Figure 2G). ITGB2 was enriched in 46 pathways, such as regulation of actin cytoskeleton (Figure 2H). MMP9 was enriched in 43 pathways, such as regulation of Fc gamma R-mediated phagocytosis (Figure 2I). TIMP1 was enriched in 25 pathways, such as oxidative phosphorylation (Figure 2J). The top 5 up- and downregulated pathways were presented (by NES value). The 4 key genes were co-replete with multiple pathways, such as circadian rhythm mammals, indicating that key genes might function together through this pathway.

### 3.3 TIMP1 gene silencing in the OA rat model was successfully constructed

**FIGURE 3:**
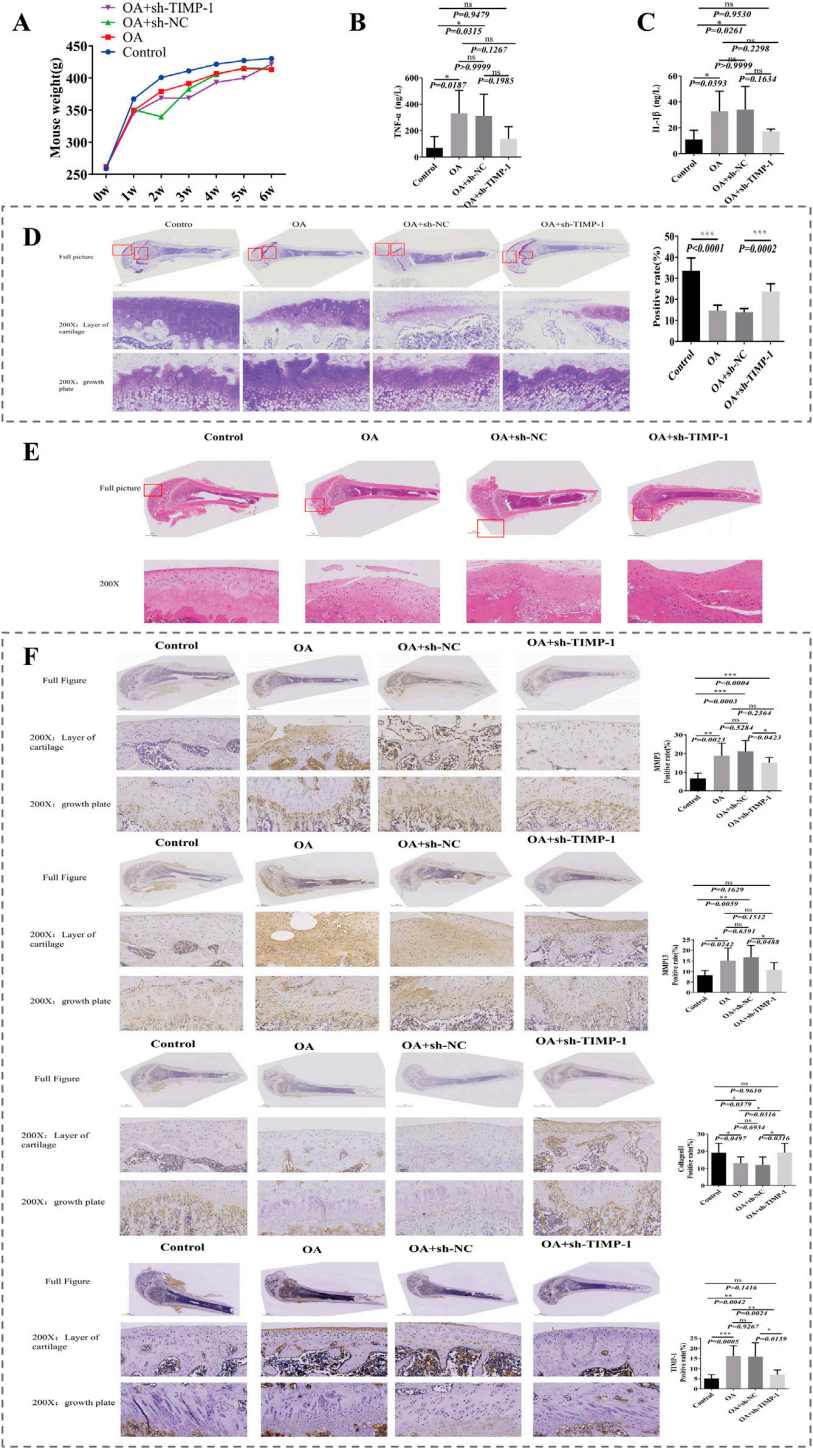
Validation of the TIMP1 gene silencing OA rat model. **(A)** Body weight changes in four groups during model construction. **(B,C)** Serum levels of TNF-α and IL-1β detected by ELISA. **(D)** Toluidine blue staining of cartilage tissue sections with semiquantitative analysis. **(E)** HE staining showing histological changes in cartilage tissue. **(F)** IHC staining and expression analysis of Collagen II, MMP3, MMP13, and TIMP1 in femoral cartilage tissues.

To explore the biological role of TIMP1 in OA, we constructed a TIMP1 gene silencing model and evaluated model through a series of experiments. During construction of animal model, body weight of control group was notably higher than that of the other three groups (*P* < 0.05) (Figure 3A). Contents of IL-1β and TNF-α in serum were detected by ELISA. Compared with the Control group, expressions of IL-1β and TNF-α in OA group were notably rose. Compared with OA + sh-NC group, expressions of IL-1β and TNF-α in OA + sh-TIMP-1 group were declined. This indicated that inflammatory response was aggravated in OA group, and low expression of TIMP1 gene would reduce inflammatory response in OA (Figures 3B,C). Toluidine blue staining demonstrated that compared with Control group, expression in OA group declined markedly. Compared with OA + sh-NC group, expression in OA + sh-TIMP-1 group rose significantly (Figure 3D), indicating that TIMP-1 gene improved condition of cartilage tissue. Meanwhile, in H&E staining, compared with Control group, cells in cartilage layer of OA group were arranged in a disordered manner, tissues were severely damaged, and the number of inflammasomes rose. Tissues in OA + rAAV-NC group were severely damaged and there was obvious infiltration of inflammatory cells. Compared with OA + rAAV-NC group, cartilage damage in OA + rAAV-TIMP-1 group was alleviated (Figure 3E). Afterwards, expression amounts of Collagen II, MMP3, MMP13 and TIMP1 gene were detected by IHC. Among them, Collagen II is a key component of cartilage tissue, and its content can directly reflect degree of cartilage damage. MMP3 and MMP13 are marker genes for degradation of cartilage matrix. Compared with control group, MMP3, MMP13, and TIMP1 were notably overexpressed while Collagen II was markedly underexpressed in OA group. Compared with OA + rAAV-NC group, expression trends of 4 genes in OA + rAAV-TIMP1 group were opposite to results in OA vs. Control comparison (Figure 3F). Above results indicated that OA model and TIMP1 gene silencing model were successfully constructed, and TIMP1 gene was a risk factor in OA (Figure 3F).

### 3.4 Expression amounts of key genes were validated via RT-qPCR

**FIGURE 4:**
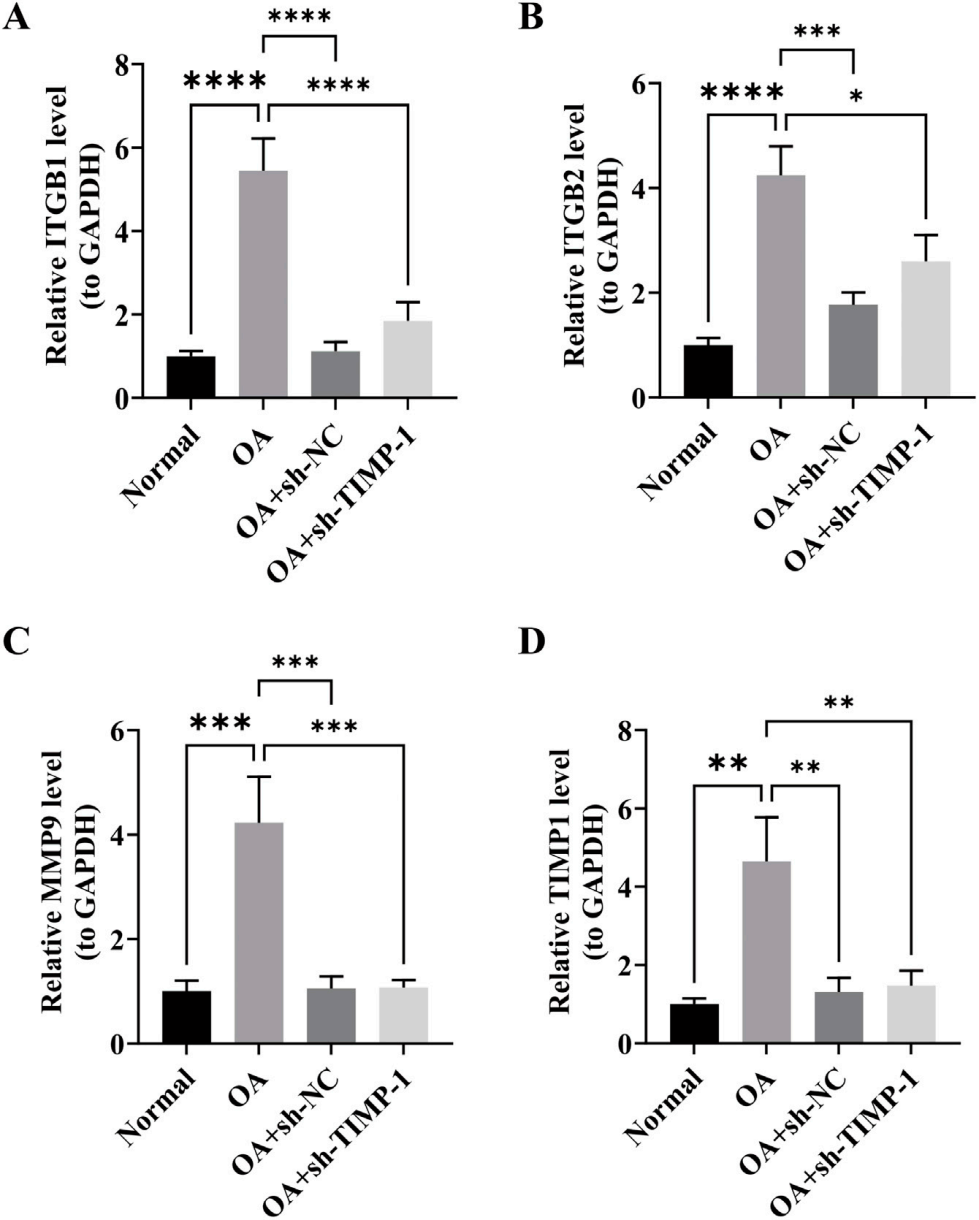
Validation of key gene expression levels by RT-qPCR. **(A–D)** Relative mRNA expression levels of ITGB1, ITGB2, MMP9, and TIMP1 in Normal, OA, OA + sh-NC, and OA + sh-TIMP1 groups *P* < 0.05 (*), *P* < 0.01 (**), *P* < 0.001 (***), *P* < 0.0001 (****).

RT-qPCR analysis revealed that, compared with the normal group, the expression levels of ITGB1, ITGB2, MMP9, and TIMP1 were all elevated in the OA group (*P* < 0.05). However, after silencing TIMP-1, the expression levels of ITGB1, ITGB2, MMP9, and TIMP1 decreased (*P* < 0.05). These results were consistent with the previous findings, indicating that TIMP1 was a risk factor for OA (Figures 4A–D).

### 3.5 There were multiple DEMs concerned with various signaling pathways

**FIGURE 5:**
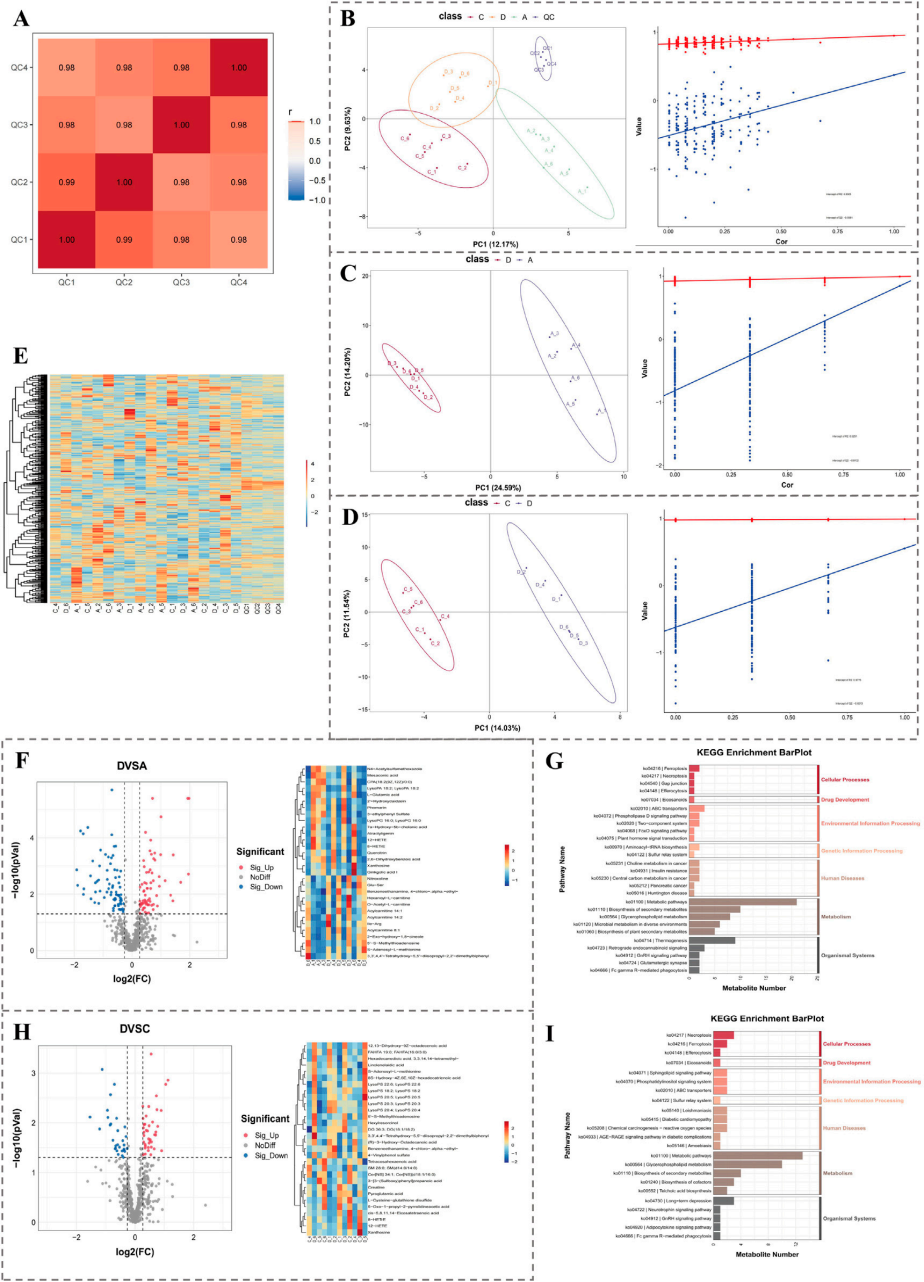
Metabolomics analysis of OA-related differential metabolites. **(A)** Pearson correlation analysis indicates sample stability. **(B–D)** PLS-DA plots and permutation tests showing group separations: D vs. A, D vs. C. **(E)** Heatmap of metabolite abundance across samples. **(F,G)** Volcano plot, clustering heatmap, and KEGG enrichment of DEMs between group D and A. **(H–I)** Volcano plot, clustering heatmap, and KEGG enrichment of DEMs between group D and C.

In light of acquired metabolomics data, the results of Pearson correlation analysis presented that the instrument was relatively stable throughout the detection process (Figure 5A). PLS-DA analysis presented that 3 groups of samples were clearly separated and could be effectively distinguished (Figure 5B). Results of the permutation test indicated that the model was not overfitted (Figure 5B). The same results of PLS-DA analysis and permutation test were attained within groups D and A (Figure 5C) and groups D and C (Figure 5D). Clustering heatmap displayed abundances of metabolites in each sample (Figure 5E). The above results demonstrated that the quality of metabolomics data was good and could be applied for subsequent analysis.

In group D and group A, 99 DEMs1 were ascertained, among which there were 46 upregulated metabolites and 53 downregulated metabolites (Figure 5F). The 99 differential metabolites were enriched in 46 pathways, such as lipid metabolism. Figure presents the top 5 secondary classification pathways (ranked by *P* value) within each KEGG first-level classification (Figure 5G).

In group D and group C, a total of 58 DEMs2 were identified. Among these DEMs2, 31 were upregulated metabolites, while 27 were downregulated ones (Figure 5H). These 58 DEMs2 were found to be replete with 31 pathways, such as cell growth and death. Moreover, within each KEGG first-level classification, the figure demonstrated the top 5 secondary classification pathways that were ranked by *P* value (Figure 5I).

### 3.6 Multiple differential microorganism genera were identified

**FIGURE 6:**
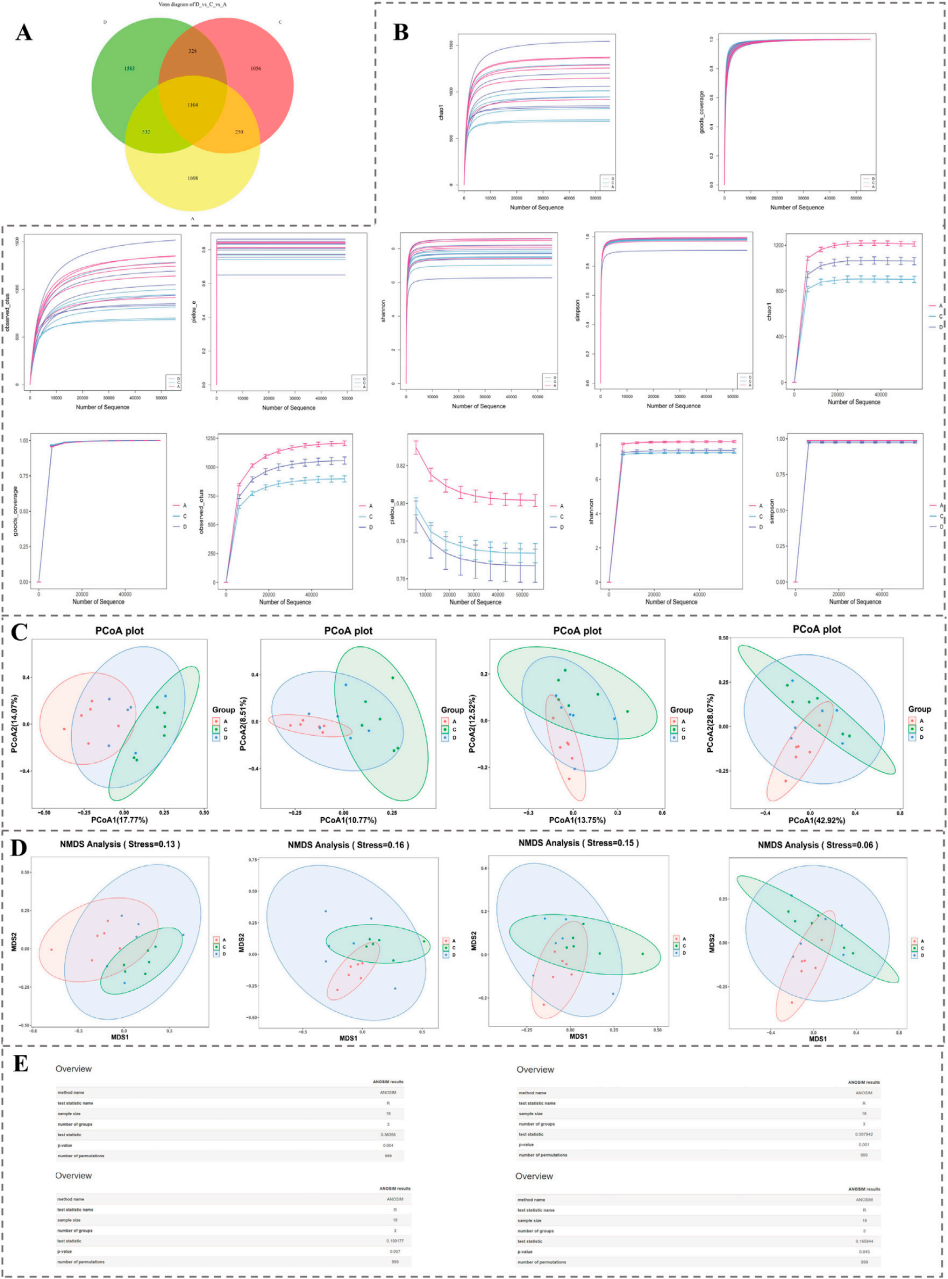
Microbiota diversity analysis based on 16S rRNA sequencing. **(A)** Venn diagram showing shared and unique ASVs among groups A, C, and D. **(B)** Rarefaction curves for alpha diversity indices, demonstrating sufficient sequencing depth. **(C)** PCoA plots illustrating distinct clustering patterns among groups. **(D)** NMDS plots showing significant compositional differences across samples (stress <0.2). **(E)** Results of Anosim analyses confirming statistical significance and group clustering reliability.

Ground on acquired 16S rRNA gene sequencing data, the number of ASVs shared by group A, group C and group D was 1,104 (Figure 6A). Rarefaction curves of various indices gradually became flat as amount of sequencing data rose, indicating that microbiome data had sufficient sequencing depth at both overall level and level of each group and could be applied for subsequent analysis (Figure 6B). Results of PCoA demonstrated that samples could be clearly distinguished (Figure 6C). In NMDS, stress values of four indicators were all under 0.2, indicating that species composition in samples was of certain significance (Figure 6D). Results of Anosim demonstrated that grouping was statistically significant (R > 0 and *P* < 0.05) (Figure 6E). Results of Adonis analysis further confirmed that grouping was reasonable (R^2^ = 1) and results had a relatively high credibility (*P* < 0.01).

**FIGURE 7:**
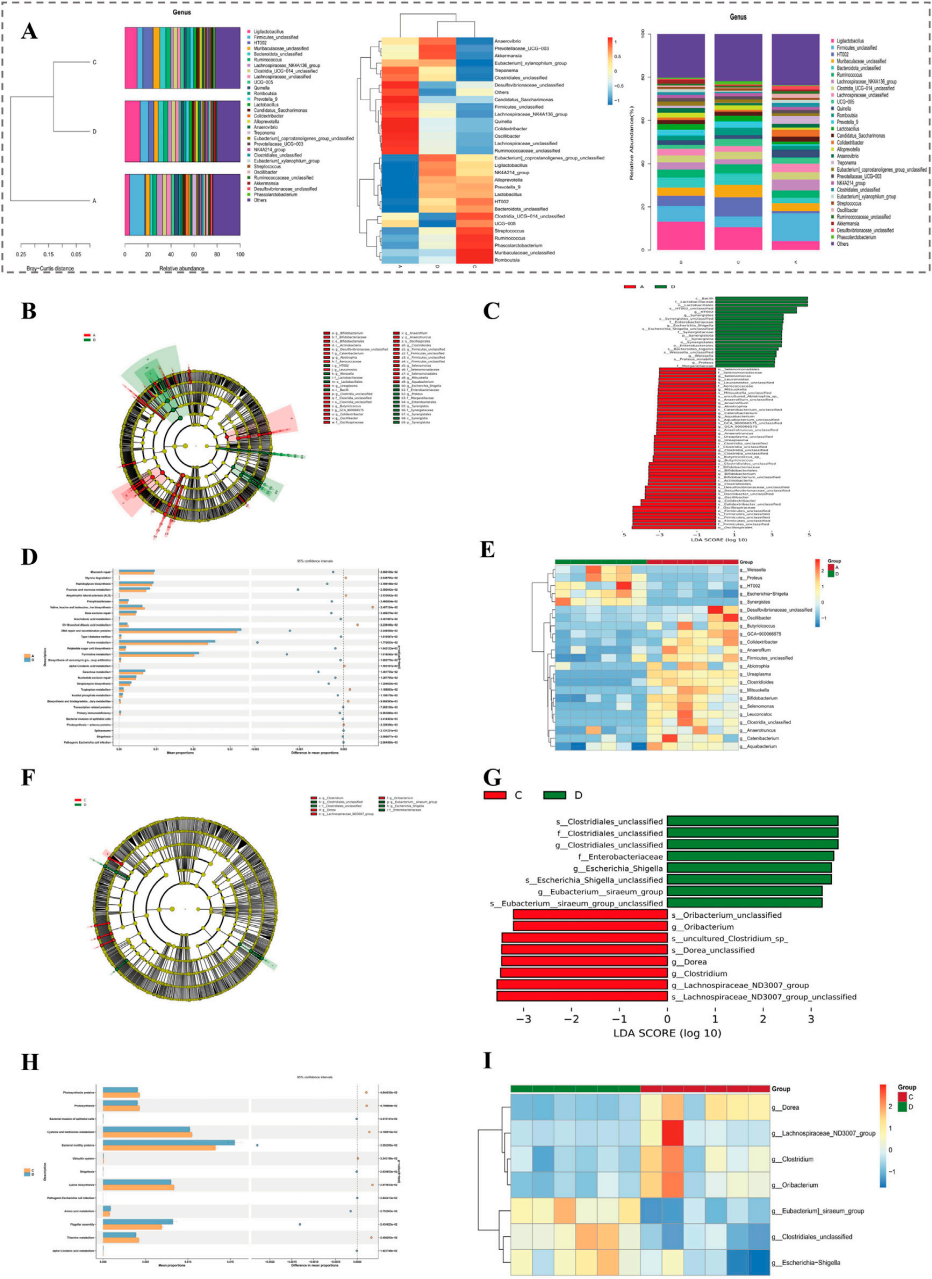
Differential microbiota and functional pathway analysis based on 16S rRNA sequencing. **(A)** Relative abundance and clustering of the top 30 genera across groups. **(B,C)** LEfSe analysis of differential microbiota between group A and D. **(D)** KEGG enrichment (tertiary level) of 69 differential microbiota between group A and D. **(E)** Heatmap of 23 significant genera in group A vs. D comparison. **(F,G)** LEfSe analysis of 16 differential genera between group D and C. **(H)** KEGG enrichment (tertiary level) of 13 pathways between group D and C. **(I)** Heatmap of 7 significant genera in group D vs. C comparison.

Species with the top 30 relative abundances at the genus level were presented in figure (Figure 7A). Among them, those with relatively high relative abundances included g ligilactobacillus, g firmicutes, and g muribaculaceae. In group A and group D, a total of 69 differential microbiota 1 were identified, among which 21 were upregulated and 48 were downregulated (Figures 7B,C). Subsequently, a total of 51 pathways were enriched at the KEGG tertiary classification level. The top 30 significant pathways were presented in a figure, such as adipocytokine signaling pathway (Figure 7D). In addition, 23 differential microbiota genera 1 were displayed through a heatmap, among which 4 were upregulated and 19 were downregulated (Figure 7E).

In both group D and group C, comprehensive analyses led to recognition of 16 differential microbiota 2. Out of these, 8 exhibited upregulated patterns while other 8 demonstrated downregulated characteristics (Figures 7F,G). Following this initial discovery, further investigations at the KEGG tertiary classification level unveiled a total of 13 pathways that were markedly enriched. The top 30 significant pathways were presented in figure, such as african trypanosomiasis (Figure 7H). In addition, 7 differential microbiota genus 2 were displayed through a heatmap, among which 3 were upregulated and 4 were downregulated (Figure 7I).

### 3.7 A total of 2021 DEGs1 and 7 DEGs1 were associated with diverse signaling pathways

**FIGURE 8:**
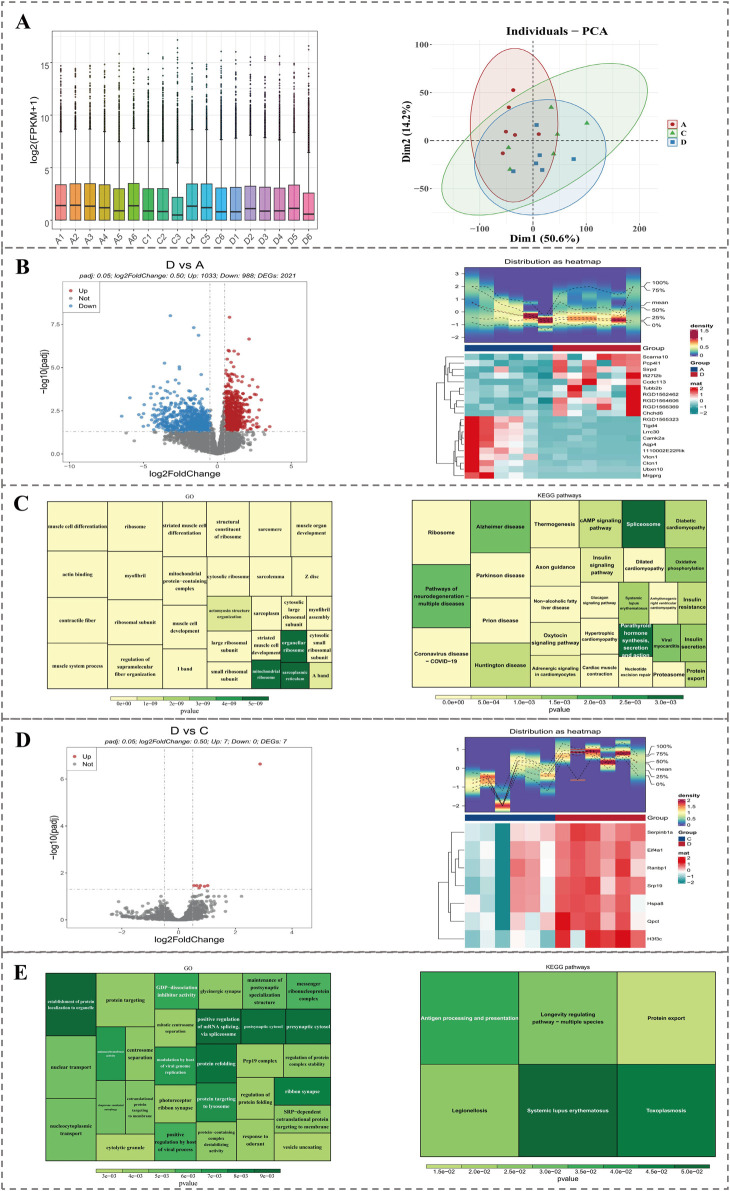
Transcriptomic analysis of DEGs between groups. **(A)** Boxplot and PCA showing expression distribution and sample clustering across groups. **(B)** Volcano plot and heatmap of the top 20 DEGs between group D and A. **(C)** GO and KEGG enrichment of DEGs1 between group D and A. **(D)** Volcano plot and heatmap of 7 DEGs2 between group D and C. **(E)** GO and KEGG enrichment of DEGs2 between group D and C.

In transcriptomics data, samples from three groups could be distinguished, and expression amounts of 18 samples were all at the same level, which could be applied for subsequent analysis (Figure 8A). In comparison within group D and group A, a total of 2,021 DEGs1 were identified. Among them, 1,033 genes were upregulated and 998 genes were downregulated. The top 10 upregulated and downregulated genes were labeled in a volcano plot, and the expression patterns of these 20 genes were displayed through a heatmap (Figure 8B). Furthermore, enrichment analysis yielded a total of 640 GO terms, comprising 416 biological processes (BPs, e.g., muscle cell development), 165 cellular components (CCs, e.g., contractile fiber), and 59 molecular functions (MFs, e.g., actin binding), in addition to 55 KEGG pathways (e.g., nucleotide excision repair). The top 30 significant GO functions and KEGG pathways were presented in figures (Figure 8C).

In comparison, within group D and group C, a total of 7 DEGs2 were identified. Among them, 7 genes were upregulated. DEGs2 were labeled in the volcano plot, and expression patterns of DEGs2 were displayed through a heatmap (Figure 8D). Furthermore, enrichment analysis yielded a total of 174 GO terms, comprising 122 biological processes (BPs, e.g., regulation of protein folding), 31 cellular components (CCs, e.g., prp19 complex), and 21 molecular functions (MFs, e.g., protein-containing complex destabilizing activity), in addition to 6 KEGG pathways (e.g., protein export). The top 30 significant GO functions and all KEGG pathways were presented in figures (Figure 8E).

### 3.8 A correlation was demonstrated within 6 biomarkers, 1 key, microorganism and 9 key metabolites

**FIGURE 9:**
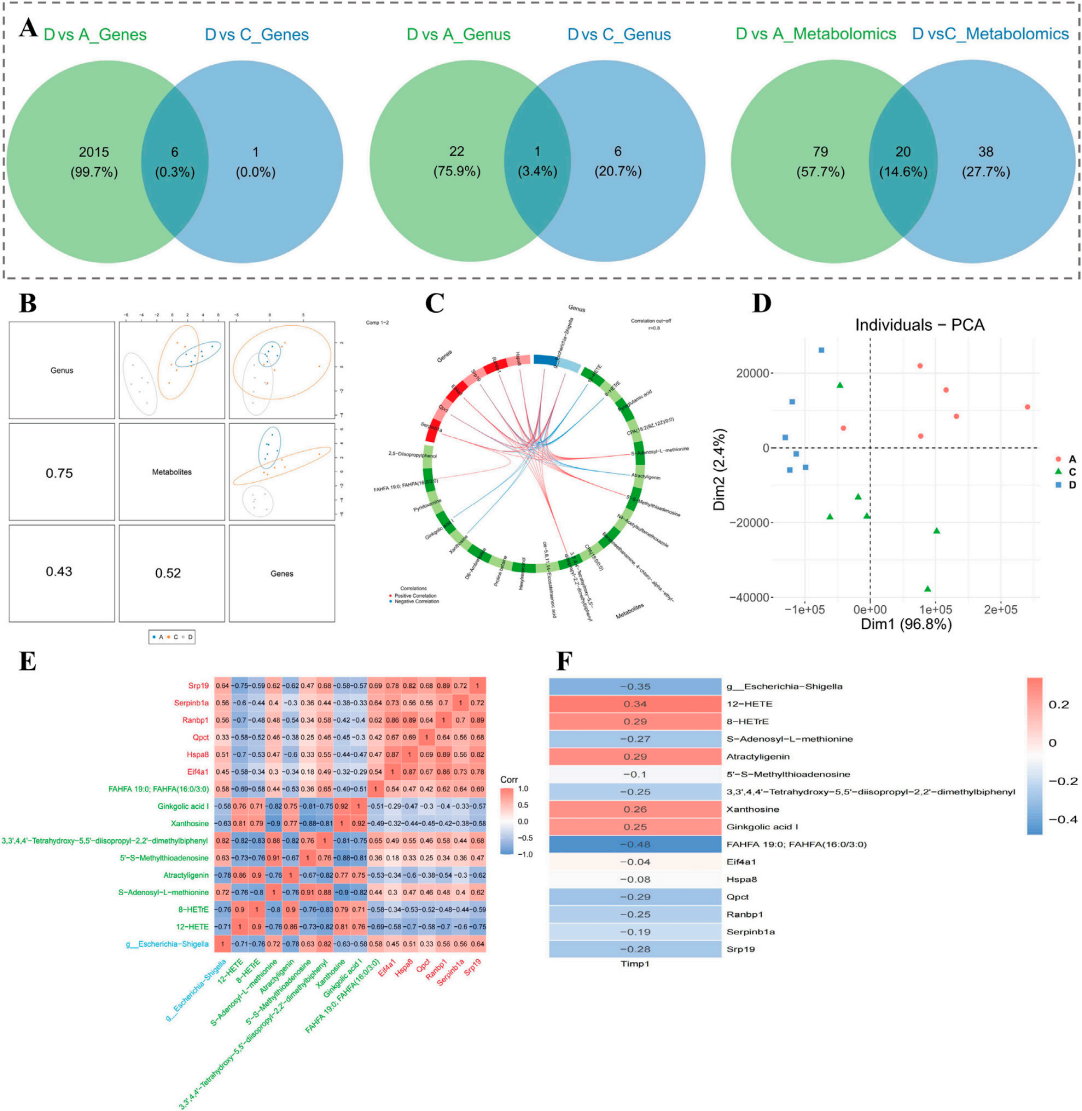
Correlation analysis of key biomarkers, microorganisms, and metabolites. **(A)** Venn diagrams showing overlap of differential genes, genera, and metabolites across comparisons. **(B)** Correlation matrix showing strongest associations between metabolites and microbial genera. **(C)** Network of 6 biomarkers, 1 key genus, and 9 key metabolites identified through integrated analysis. **(D)** PCA based on combined expression profiles of biomarkers, genus, and metabolites. **(E)** Correlation heatmap of top key variables; red and blue indicate positive and negative correlations, respectively. **(F)** TIMP1-associated correlations with key metabolites and genus, ranked by coefficient strength.

A total of 6 candidate biomarkers, 1 candidate microbial genus, and 20 candidate metabolites were acquired (Figure 9A). Through correlation analysis, candidate metabolites had the highest correlation with candidate microbial communities overall (cor = 0.75, *P* < 0.05) (Figure 9B). At the same time, 6 biomarkers, 1 key microbial genus, and 9 key metabolites were identified (Figure 9C). Results of PCA indicated that expressions of biomarkers, key microbial genera, and key metabolites were specific among the three groups (A, C, and D) (Figure 9D). In addition, there was a conspicuous positive correlation within key metabolites and biomarkers (cor >0.3, *P* < 0.05) (Figure 9E). Xanthosine and Ginkgolic acid I demonstrated the highest apparent positive correlation (cor = 0.92, *P* < 0.05), while Xanthosine and 5′-S-Methylthioadenosine presented the lowest marked negative correlation (cor = −0.88, *P* < 0.05) (Figure 9E). TIMP1 revealed the lowest marked negative correlation with FAHFA 19:0; FAHFA (16:0/3:0) (cor = −0.48, *P* < 0.05), and the highest marked positive correlation with 12-HETE (cor = 0.34, *P* < 0.05) (Figure 9F).

## 4 Discussion

OA is a leading cause of disability worldwide, yet current therapies mainly offer symptomatic relief and fail to prevent disease progression. NETs, initially recognized as antimicrobial structures, have recently been implicated in chronic inflammation and tissue degradation, including in joint diseases, both of which are hallmarks of OA. While the precise contribution of NETs to OA remains elusive, their links to immune activation and ECM breakdown suggest a potential pro-degenerative role (Delgado-Rizo et al., 2017). In this study, we focused on the NET-associated gene TIMP1 by combining transcriptomic data mining with *in vivo* gene silencing in an OA rat model. Through multi-omics approaches—including transcriptomics, metabolomics, and 16S rRNA microbiome profiling—we identified key genes, metabolites, and microbial taxa influenced by TIMP1 expression. These results suggest that TIMP1 may function as a central node in the immunometabolic network, bridging ECM remodeling, inflammation, and gut–joint crosstalk, offering new insights into OA pathophysiology. Notably, prognostic work in ACL injury has linked clinical and MRI features to long-term outcomes, underscoring the value of coupling molecular markers with imaging/clinical indicators when constructing predictive models (Romero et al., 2021). These mechanistic insights not only expand our understanding of OA immunometabolic networks but also provide a theoretical foundation for future pharmacological investigations, including virtual drug screening, molecular docking, and the development of TIMP1- or NETs-targeted therapies.

TIMP1 (Tissue Inhibitor of Metalloproteinases 1) is a secreted glycoprotein of the TIMP family, primarily responsible for regulating extracellular matrix (ECM) turnover by inhibiting matrix metalloproteinases (MMPs). Structurally, it contains two functional domains: an N-terminal domain that directly inhibits MMPs, and a C-terminal domain involved in cell survival and signaling (Zhang et al., 2024). In OA, TIMP1 plays a paradoxical role. On one hand, it inhibits MMPs such as MMP9 and MMP13 to prevent excessive ECM degradation, thereby preserving cartilage structure (Jayadev et al., 2020). On the other hand, persistent overexpression may impair matrix turnover and induce synovial fibrosis and joint stiffness—hallmarks of late-stage OA (Wei et al., 2021; Zhang et al., 2019). In our study, TIMP1 was markedly upregulated in OA tissues and identified as a NETs-associated gene. *In vivo* silencing of TIMP1 significantly reduced IL-1β, TNF-α, MMP3, and MMP13 levels, and ameliorated histopathological damage, supporting its pathological role in OA. Additionally, persistent TIMP1 secretion by mesenchymal stem cells (MSCs)—independent of their differentiation state—has been shown to maintain trophic repair functions, further emphasizing its role in inflammation and tissue remodeling (Salerno et al., 2020). Mechanistic studies also reveal that TIMP1 can promote NET formation through signaling in neutrophils, independent of MMP inhibition (Schoeps et al., 2021). Thus, TIMP1 emerges not only as an ECM regulator but also as an upstream inducer of inflammation. Our findings support the view that TIMP1 contributes to OA through both structural disruption and immune activation, making it a promising target for therapeutic intervention.

The gut microbiota has emerged as a key modulator of OA through mechanisms involving systemic inflammation, immune priming, and metabolic reprogramming—collectively known as the “gut–joint axis” (Yang et al., 2025). In our study, 16S rRNA sequencing revealed distinct microbial compositions between OA and TIMP1-silenced groups. Functional predictions based on KEGG enrichment showed significant involvement of pathways related to adipocytokine signaling and lipid metabolism. Adipocytokines such as leptin, adiponectin, and resistin are critical mediators linking metabolic state with inflammatory responses, directly affecting chondrocyte catabolism, immune cell infiltration, and ECM turnover (Turpin et al., 2023). Of particular interest, we observed enrichment of Muribaculaceae—a genus known for butyrate production—in TIMP1-silenced rats. Butyrate has been shown to promote granzyme B expression in IL-10-producing Th1 cells via HDAC inhibition and GPR43 signaling, enhancing regulatory T cell responses and reducing intestinal inflammation (Yang et al., 2024). These findings suggest that butyrate may exert systemic anti-inflammatory effects, potentially extending to joint tissues. In line with this, the present review highlights that gut microbiota–derived short-chain fatty acids, particularly butyrate, can suppress osteoclast differentiation and regulate Th17/Treg immune balance, thereby attenuating systemic inflammation and contributing to the mitigation of OA-related pain and structural deterioration (Meléndez-Oliva et al., 2025). In parallel, our results align with prior reports that microbial SCFAs regulate host lipid metabolism, thereby influencing chondrocyte energy homeostasis and synovial inflammation (Xu et al., 2024). Altogether, these observations imply that gut microbial modulation of adipocytokine and lipid-related pathways—potentially mediated by SCFAs like butyrate—could represent a mechanistic link between TIMP1 suppression and improved OA outcomes. Beyond intrinsic molecular and microbial mechanisms, external environmental influences also shape musculoskeletal pain trajectories. A 14-year retrospective cohort study in Spanish primary care demonstrated that climatic variables, particularly temperature and barometric pressure, significantly impacted chronic musculoskeletal pain referrals, underscoring the broader interplay between biological regulation and environmental stressors in modulating pain perception and healthcare demand (Zaldívar et al., 2025). Moreover, KEGG pathway enrichment identified 13 significantly altered pathways between the TIMP1-silenced and OA control groups (Group D vs. C), including lysine biosynthesis, amino sugar metabolism, and immune-related signaling. Although these pathways differ from those altered in the D vs. A comparison, their distinctiveness highlights the specific regulatory impact of TIMP1 silencing on microbial function and its potential to rebalance host metabolic and immune homeostasis. Metabolomic profiling identified nine TIMP1-associated differential metabolites, primarily enriched in pathways related to lipid metabolism, nucleotide turnover, and immune signaling—all closely linked to OA progression. Recent studies underscore the pathological relevance of these pathways; mitochondrial oxidative stress has been shown to trigger excessive formation of NETs, thereby aggravating vascular inflammation and tissue injury in aged models (Wang et al., 2017). Similarly, altered extracellular nucleotide metabolism may enable pathogens to evade NET-mediated clearance, a mechanism that could be co-opted in OA to modulate local immune activation (Afonso et al., 2021). These findings suggest that TIMP1 may contribute to OA not only by modulating ECM structure, but also through systemic immunometabolic disruption. Among these, fatty acid esters of hydroxy fatty acids (FAHFAs) tended to decrease in OA rats and were relatively increased after TIMP1 silencing. FAHFAs are known to enhance insulin sensitivity and suppress adipose inflammation by promoting glucose uptake and attenuating pro-inflammatory cytokine production via the IRF3–AIG1 axis (Yan et al., 2024). Their reduction may reflect a pro-inflammatory, insulin-resistant state associated with high TIMP1 expression. In contrast, 12-HETE—a pro-inflammatory metabolite derived from arachidonic acid—showed an opposite trend, being more abundant in OA tissues and reduced upon TIMP1 knockdown. 12-HETE has been implicated in activating p38 MAPK and NF-κB pathways, thereby promoting immune cell recruitment and tissue degradation in both vascular and articular contexts (Chen et al., 2024). This inverse regulation of FAHFAs and 12-HETE suggests that TIMP1 may skew lipid metabolism toward a catabolic and inflammatory profile. These findings may hold translational significance. Restoration of FAHFAs—a lipid class with anti-inflammatory and insulin-sensitizing properties—may represent a metabolic biomarker indicative of therapeutic response in osteoarthritis. Conversely, increased levels of 12-HETE, a pro-inflammatory lipid mediator, could serve as a predictor of disease activity or treatment resistance. The genus Muribaculaceae, enriched following TIMP1 silencing and known for its capacity to produce butyrate and promote immune tolerance, may offer a novel microbial target for modulating the gut–joint axis in osteoarthritis management (Liu et al., 2025). Such molecular candidates may ultimately support precision medicine strategies in OA, enabling earlier diagnosis, stratified treatment, and monitoring of therapeutic efficacy (Zhai, 2021). Integration of such markers into clinical workflows—particularly through non-invasive serum or microbiome profiling—could help tailor interventions based on individual metabolic or microbial profiles. In addition to lipid- and microbiota-related candidates, other classes of metabolites identified in our multi-omics analysis may also hold clinical value. Nucleotide- and methylation-related compounds, such as xanthosine and 5′-S-methylthioadenosine (MTA), may reflect systemic metabolic states that influence inflammation and tissue remodeling in OA. These metabolites are not only mechanistically linked to oxidative stress and immune signaling, but may also serve as emerging molecular indicators of disease severity or progression. Consistent with this, we also observed significant alterations in nucleotide- and methylation-related compounds, including xanthosine and 5′-S-methylthioadenosine (MTA). MTA, a byproduct of microbial methionine metabolism and salvage pathways, suppresses PRMT5-mediated arginine methylation and regulates lipid metabolism and insulin signaling. Its depletion in OA may contribute to epigenetic dysregulation and foster a fibrotic, pro-inflammatory microenvironment (Lyu et al., 2024). Collectively, our data indicate that TIMP1 modulates OA development through coordinated metabolic reprogramming, affecting inflammation, tissue remodeling, and oxidative stress responses.

Transcriptomic profiling identified two distinct sets of DEGs: 2021 genes associated with OA pathology (DEGs1), and 7 genes modulated by TIMP1 silencing (DEGs2). Enrichment analysis of DEGs1 indicated strong involvement of extracellular matrix (ECM)–receptor interaction and focal adhesion pathways, both critical for chondrocyte–matrix communication, mechanotransduction, and structural maintenance of the joint. Disruption of these pathways is a well-recognized driver of cartilage degradation and biomechanical dysfunction in OA (Chun et al., 2024; Yao et al., 2023). TIMP1 itself, as a known ECM regulator, may disturb the fine balance between matrix synthesis and degradation when overexpressed, thereby exacerbating joint damage. In addition to matrix-related pathways, nucleotide excision repair emerged as a top-enriched category, likely reflecting increased DNA repair activity in response to oxidative stress and genotoxic damage in OA chondrocytes (Ni et al., 2023). Ribosomal and translational pathway enrichment may further suggest heightened protein synthesis demand during tissue remodeling. In contrast, analysis of DEGs2—genes responsive to TIMP1 silencing—highlighted enrichment in pathways related to protein processing in the endoplasmic reticulum and RNA splicing (including the prp19 complex), with possible involvement in lysosomal and post-transcriptional regulation. These pathways are vital for maintaining proteostasis and RNA quality control under metabolic stress. Their dysregulation has been linked to enhanced ER stress, chondrocyte apoptosis, and OA progression (Kim et al., 2010; Wang et al., 2022). Together, these transcriptomic data suggest that TIMP1 exerts a broad regulatory influence—extending beyond ECM remodeling to include DNA repair, protein homeostasis, and RNA processing—underscoring its central role in coordinating cellular stress responses in OA.

Through multi-omics integration, we identified a core set of six potential biomarkers, nine differential metabolites, and one key microbial genus—Muribaculaceae—that were significantly associated with TIMP1 expression and OA progression. These results collectively reflect the multifactorial nature of OA and highlight the regulatory influence of TIMP1 across molecular, metabolic, and microbial domains. Beyond molecular and microbial signatures, functional markers also merit attention. A recent meta-analysis showed that quantitative sensory testing (QST) can stratify OA patients by pain and disability outcomes, underscoring the translational value of combining patient-centered functional assessments with molecular findings (Murphy et al., 2025). Among the candidate biomarkers, SPP1 (osteopontin) and A2M (alpha-2-macroglobulin) are particularly notable. SPP1 acts as a pro-inflammatory mediator that promotes macrophage infiltration and MMP expression in synovial tissues, exacerbating joint damage. In contrast, A2M has been proposed as a disease-modifying OA therapy due to its ability to inhibit proteolytic enzymes and inflammatory cytokines (Dai et al., 2023; Zheng et al., 2023). The differential metabolites—especially FAHFAs, 12-HETE, MTA, and xanthosine—represent central metabolic nodes involved in lipid signaling, oxidative stress, and nucleotide metabolism. Of these, FAHFAs and MTA, which are rarely reported in OA literature, emerged as potential anti-inflammatory mediators suppressed in high-TIMP1 states, indicating their novelty and potential as diagnostic markers (Wu et al., 2020; Tang et al., 2022). The genus Muribaculaceae, known for producing short-chain fatty acids and modulating regulatory T cells, was enriched in TIMP1-silenced rats, suggesting a protective, microbiota-mediated immunoregulatory role (Zhu et al., 2024). Conversely, Escherichia-Shigella was the most TIMP1-associated genus in the integrated network, correlating with pro-inflammatory metabolites and supporting its link to gut-derived systemic inflammation and OA exacerbation (Wang et al., 2021). Recent multi-omics studies in other inflammatory diseases support our findings. In colorectal cancer, TIMP1 was shown to promote disease progression by modulating ferroptosis, immune infiltration, and redox-related metabolism (Jin et al., 2025). Similarly, in lung adenocarcinoma, NET-associated multi-omics signatures—also involving TIMP1—were linked to metabolic remodeling and immunosuppression (Shan et al., 2025). These results highlight TIMP1’s shared role in neutrophil activation and immune–metabolic dysregulation across diverse pathologies, echoing its function in OA. These results highlight TIMP1’s shared role in neutrophil activation and immune–metabolic dysregulation across diverse pathologies, echoing its function in OA. Altogether, these integrated findings position TIMP1 as a central hub orchestrating cross-talk between immune signaling, metabolic remodeling, and microbial dysbiosis—uncovering novel therapeutic and diagnostic targets in OA. This perspective resonates with frailty research, where aging-related vulnerability is defined as a multidimensional syndrome shaped by systemic inflammation and tissue remodeling. Such holistic frameworks support our systems biology approach and emphasize the need for multi-level strategies in OA (Fernández-Carnero et al., 2024). Recent advances further indicate that machine learning–based systemic approaches can optimize frailty detection, a condition closely linked with musculoskeletal decline, thereby positioning OA research within the broader landscape of precision and predictive medicine (Fernández-Carnero et al., 2025).

In summary, this study identifies TIMP1 as a pivotal immunometabolic regulator in osteoarthritis (OA), orchestrating extracellular matrix remodeling, inflammatory signaling, metabolic reprogramming, and gut microbiota dynamics. By integrating transcriptomic, metabolomic, and microbial profiling with *in vivo* gene silencing, we provide compelling evidence that TIMP1 serves as a molecular hub linking structural damage with systemic dysregulation, thereby offering novel avenues for disease stratification and targeted intervention in OA. Nevertheless, several limitations should be acknowledged. First, reliance on a rat model may limit translational relevance due to interspecies differences in gene expression and immune–microbiota interactions. Second, the multi-omics associations identified here are primarily correlative; causal relationships among TIMP1, metabolic alterations, and microbial shifts remain to be experimentally validated. Third, the relatively small sample size may affect the generalizability of certain findings. Future work should include validation in larger, well-characterized human cohorts, along with advanced systems such as organoids or humanized models, to clarify mechanistic pathways and assess their value as biomarkers or therapeutic targets. Although TIMP1 was identified as a NETs-associated gene, the present study did not directly quantify NETs formation. Future investigations should address this gap to establish a more complete causal link between TIMP1 and NET biology in OA.

## 5 Conclusion

This study highlights TIMP1 as a central immunometabolic regulator in osteoarthritis, integrating extracellular matrix remodeling with inflammatory signaling, metabolic reprogramming, and gut microbiota alterations. Through multi-omics analysis and *in vivo* validation, we reveal a TIMP1-centered network of biomarkers, metabolites, and microbial taxa that collectively drive OA progression. These findings not only deepen our mechanistic understanding of OA but also provide a pharmacological basis for targeting TIMP1-associated pathways, supporting the development of precision diagnostics and novel disease-modifying therapies.

## Data Availability

The datasets generated and analyzed in this study are available within the article and its Supplementary Material. Public transcriptomic datasets were obtained from GEO (GSE114007 and GSE57218). Additional data supporting the findings of this study are available from the corresponding author upon reasonable request.

## Abbreviations

16S rRNA, 16S ribosomal RNA; ACL, Anterior cruciate ligament; AUC, Area under the curve; ASV, Amplicon sequence variant; DEGs/DEMs, Differentially expressed genes/metabolites; DNA, Deoxyribonucleic acid; ECM, Extracellular matrix; ELISA, Enzyme-linked immunosorbent assay; FAHFAs, Fatty acid esters of hydroxy fatty acids; FC/log2FC, Fold change/log2 fold change; FDR, False discovery rate; GEO, Gene Expression Omnibus; GO, Gene Ontology; GSEA, Gene Set Enrichment Analysis; H&E, Hematoxylin and eosin; IHC, Immunohistochemistry; KEGG, Kyoto Encyclopedia of Genes and Genomes; LC–MS/MS, Liquid chromatography–tandem mass spectrometry; LEfSe/LDA, Linear discriminant analysis effect size/Linear discriminant analysis; MMP, Matrix metalloproteinase; MPO, Myeloperoxidase; NETs, Neutrophil extracellular traps; NMDS/PCoA, Non-metric multidimensional scaling/Principal coordinates analysis; OA, Osteoarthritis; OTU, Operational taxonomic unit; PCA, Principal component analysis; PLS-DA/VIP, Partial least squares discriminant analysis/Variable importance in projection; PPI, Protein–protein interaction; QC, Quality control; ROC, Receiver operating characteristic; RT-qPCR, Reverse transcription quantitative PCR; shRNA, Short hairpin RNA; TIMP1, Tissue inhibitor of metalloproteinases-1; UPLC–MS, Ultra-performance liquid chromatography–mass spectrometry.
